# The TRH test provides valuable information in the diagnosis of central hypothyroidism in patients with known pituitary disease and low T4 levels

**DOI:** 10.3389/fendo.2023.1226887

**Published:** 2023-10-02

**Authors:** Sara Ellegaard Christensen, Liv Norma Smith, Christian Alexander H. Rosendal, Helga Angela Gulisano, Kåre Schmidt Ettrup, Peter Vestergaard, Eigil Husted Nielsen, Jesper Scott Karmisholt, Jakob Dal

**Affiliations:** ^1^ Department of Endocrinology, Aalborg University Hospital, Aalborg, Denmark; ^2^ Department of Neurosurgery, Aalborg University Hospital, Aalborg, Denmark; ^3^ Steno Diabetes Centre North Jutland, Aalborg, Denmark

**Keywords:** thyrotropin-releasing hormone (TRH), pituitary disease, central hypothyroidism, TRH test, hypopituitarism

## Abstract

**Objective:**

To evaluate the value of the thyrotropin-releasing hormone (TRH) test in the diagnosis of central hypothyroidism (CH) in patients with pituitary disease.

**Methods:**

Systematic evaluation of 359 TRH tests in patients with pituitary disease including measurements of thyroxine (T4), TBG-corrected T4 (T4_corr_), baseline TSH (TSH_0_) and relative or absolute TSH increase (TSH_fold_, TSH_absolute_).

**Results:**

Patients diagnosed with CH (n=39) show comparable TSH_0_ (p-value 0.824) but lower T4_corr_ (p-value <0.001) and lower TSH increase (p-value <0.001) compared to patients without CH. In 54% (42 of 78 cases) of patients with low T4_corr_, the CH diagnosis was rejected based on a high TSH_fold_. In these cases, a spontaneous increase and mean normalization in T4_corr_ (from 62 to 73 nmol/L, p-value <0.001) was observed during the follow-up period (7.6 ± 5.0 years). Three of the 42 patients (7%) were started on replacement therapy due to spontaneous deterioration of thyroid function after 2.8 years. Patients diagnosed with CH reported significantly more symptoms of hypothyroidism (p-value 0.005), although, symptoms were reported in most patients with pituitary disease. The TRH test did not provide clinical relevant information in patients with normal T4 or patients awaiting pituitary surgery (78%, 281 of 359). There were only mild and reversible adverse effects related to the TRH test except for possibly one case (0.3%) experiencing a pituitary apoplexy.

**Conclusion:**

The TRH test could be reserved to patients with pituitary disease, low T4 levels without convincing signs of CH. Approximately 50% of patients with a slightly decreased T4 were considered to have normal pituitary thyroid function based on the TRH test results.

## Introduction

Central hypothyroidism is a rare condition caused by pathology in the pituitary gland and/or the hypothalamus. CH accounts for approximately 1% of all cases with hypothyroidism (1, 2) and is caused by inadequate stimulation by thyroid-stimulating hormone (TSH) of an otherwise healthy thyroid gland. Hence, the condition is often diagnosed in patients with known pituitary disease (3). Different mechanisms may lead to CH, such as a defect in TSH secretion, a reduction in the mass of thyrotrope cells (4), or reduced TSH bioactivity (1, 5, 6), all leading to a reduction of the quantity or quality of circulating TSH (7). In contrast to primary hypothyroidism, TSH and T4 levels do not correlate (5) and due to the varying bioactivity of TSH, normal or even high values of TSH are observed in some patients with CH (6). The diagnosis of CH is therefore challenging and typically based on a low T4 level in conjunction with a low, normal, or mildly elevated TSH in the setting of pituitary disease (5).

TSH is the major regulator of thyroid hormone synthesis and secretion and TSH is regulated by thyrotropin-releasing hormone (TRH), secreted from the hypothalamus (7). Until the introduction of the modern ultra-sensitive TSH assays (8, 9), the TRH test was a widely used diagnostic tool in the investigation of both thyroid function and TSH reserve. The value of the TRH test in the diagnosis of or screening for CH is however controversial and defies general recommendation (5, 8). This is however based on few and relatively small studies focusing on a screening approach for CH (5, 8, 10). Symptoms of CH are similar to those of primary hypothyroidism e.g., tiredness, sensitivity to cold, weight gain and constipation (1, 2, 11), a clinical presentation that may overlap with symptoms of other pituitary hormone deficiencies, thus further complicating the diagnostic process (2, 12).

Since all pituitary stimulation tests performed for the hypopituitarism diagnosis at our pituitary referral center have routinely included a TRH test, we aimed to examine the value of such TRH testing in the diagnosis of CH in patients with known pituitary disease.

## Methods

### TRH test procedure

The TRH test was routinely performed after a standardized protocol accompanied by either a short Adrenocorticotropic Hormone (ACTH) stimulation test (SAT) or an insulin tolerance test (ITT). Patients were admitted for overnight fasting, and the TRH stimulation test (200 μg intravenously) was performed at 9:00 a.m. under resting conditions. Baseline blood samples included total T4, thyroid binding globulin (TBG), TBG-corrected thyroxine (T4_corr_), TSH (TSH_0_), and TRH stimulated TSH after 30 minutes (TSH_30_, TSH_absolute_, TSH_fold_). Formula for calculating TBG-corrected T4 is T4_corr_ = Total T4 * (19.1 mg/L (13))/TBG.

Following references values were applied for TSH_0_ (0.3-4.5 mlU/L) and T4 (60-140 nmol/L), T4_corr_ (70-140 nmol/L), TSH_fold_ (>3) and analyzed using the Electro-Chemi Luminescence ImmunoAssay method.

Assay characteristics for TSH and total T4 were as follow (inter-assay coefficient of variation (CV)/lower detection limit): TSH, 2,7%/0.005 mIU/L; Total T4, 4.3%/5.0 nmol/L (Elecsys, Roche Diagnostics, Basel Switzerland). From February 1^st^ 2022 and onward: Alinity, Abbott Molecular Diagnostics, Des Plaines, IL, USA), were used with characteristics for TSH, 1.5%/0.0026 mIU/L and for total T4, 4.4%/7.1 nmol/L. TBG was measured with radioimmunoassays (RIA, Brahms Biotech GmbH, Henningsdorf, Germany), With inter-assay CV of 3.5% and lower detection limit of 5 mg/l. This was measured at the department of clinical biochemistry, Aalborg University Hospital (AalborgUH) under the International Organization for Standardization (ISO) standard ensuring regular quality protocol.

### Study population

A total of 359 patients with pituitary diseases who underwent TRH testing were included in the study. Only one TRH test was selected for each person, being either the first TRH test performed or the TRH test that resulted in the start of thyroid replacement therapy. Information regarding age, sex, pituitary disease, possible medical or surgical treatment for pituitary disease, TRH test results and side effects of the TRH test were included. Patients were selected from a cohort of 429 patients that were followed at the department of endocrinology at AalborgUH during a 5 year-period (2018–2022) and who underwent TRH testing. Seventy TRH tests were excluded due to either missing data (n=16) or known primary thyroid disease (n=54). Patients were grouped according to the diagnosis of CH. Group 1 includes patients diagnosed with CH who were started on thyroid replacement therapy. Group 2 included persons who were not started on T4 replacement therapy, either because they were considered to have normal pituitary-thyroid function or because they were awaiting intended tumor reducing treatment for their pituitary disease; i.e. pituitary surgery or medical therapy. In each group, subgroups A and B were defined according to T4_corr_ level (<70 nmol/L vs. ≥70 nmol/L, respectively). A total of four cases were excluded from the subgrouping, 3 patients due to incomplete data, and 1 patient who declined thyroid replacement treatment. For group 1A, 2A and 2B symptoms of hypothyroidism were retrieved from patient charts which included the following symptoms: tiredness, sensitivity to cold, weight gain, and constipation. For groups 2A and 2B follow-up measurements of T4_corr_ were collected one year after the TRH test was performed (t2) and at the patient’s last available follow-up (t3).

### Statistics

Normality of data was tested using QQ-plots, histograms and Kolmogorov-Smirnov or Shapiro Wilk depending on sample size. Normally distributed data including T4 and T4_corr_ are expressed as mean with standard deviation (SD). Changes in or between groups were tested using paired or unpaired student’s t-test, respectively. Non-normally distributed data included TSH_0_, TSH_30_, TSH_absolute_ (TSH_30_-TSH_0_), and TSH_fold_ (TSH_30_/TSH_0_) and are expressed as median and interquartile range and tested using Mann Whitney’s U-test. Patient characteristics and symptoms between groups were tested using the Chi square test. Receiver operating characteristic (ROC) curve was used to estimate the sensitivity and specificity of TSH_fold_, TSH_absolute_, T4 and T4_corr_. A significance level of <0.05 was used.

## Results

**Figure 1:**
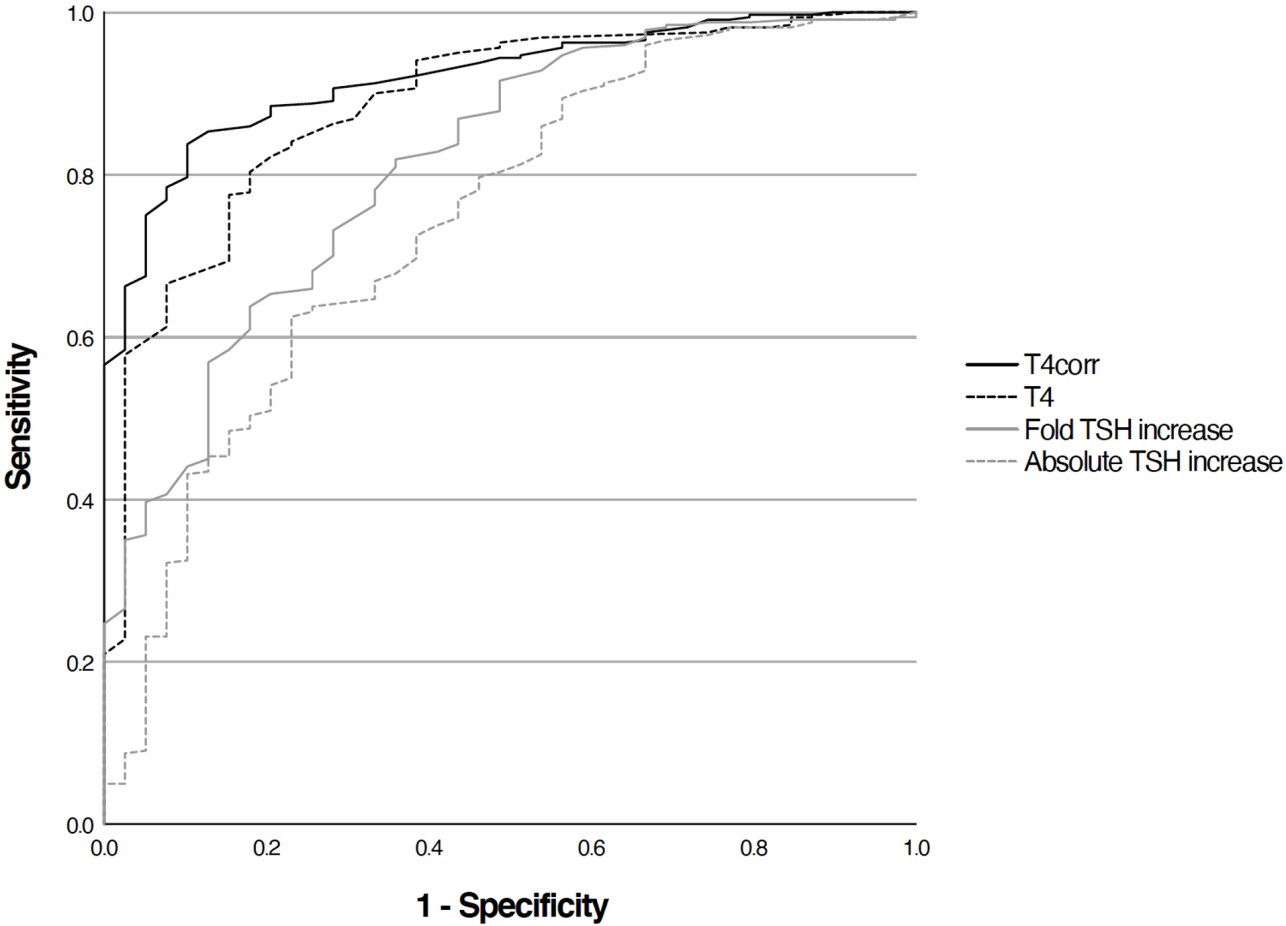
ROC-curve analysis of T4(AUC 0.88), TBG-corrected T4 (AUC 0.92), fold TSH icrease (AUC 0.81) and absolute TSH increase (AUC 0.74).

A total of 359 TRH tests were included in this study. Based on the ROC curve analysis, the TSH_fold_ measure showed a higher area under the curve (CH_AUC_) of 0.81 for the diagnosis of CH compared to the CH_AUC_ for the TSH_absolute_ (CH_AUC_ =0.74). T4_corr_ had a higher CH_AUC_ of 0.92 compared to T4 = 0.88 (**Figure 1**).

**Figure 2:**
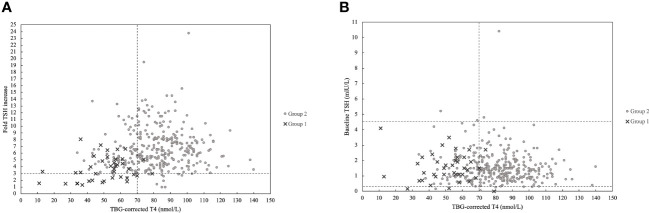
**(A)** Patients plotted according to TBG-corrected T4 and fold TSH increase with dashed line marking the lower limit of the normal range, 70 nmol/L for TBG-corrected T4 and 3 mlU/L for fold TSH increase. Group 1(n=39) is marked with crosses and represents the patients who were subsequently started in thyroid hormone replacement therapy after a TRH test and group 2 (n=320) is marked with grey dots and represents the patients who did not start thyroid hormone replacement therapy subsequently after a TRH test. **(B)** Patients plotted according to TBG-corrected T4 and baseline TSH with dashed line marking the lower limit of the normal range of 70 nmol/L for TBG-corrected T4 and the normal range of 0.3-4.5 mlU/L for baseline TSH Group 1 (n=39) is marked with crosses and represents the patients who were subsequently started in thyroid hormone replacement therapy after a TRH test and group 2 (n=320) is marked with grey dots and represents the patients who did not start thyroid hormone replacement therapy subsequently after a TRH test.

Based on the diagnosis of CH, patients were divided into group 1 diagnosed with CH (n=39) and group 2 with patients without a current need of thyroid replacement therapy (n=320, **Figures 2A, B**). There were significantly more males in group 1 (69.2%) compared to group 2 (45.3%, p-value 0.005, **Table 1**). In group 1, a higher percentage of patients received pituitary hormone replacement therapy before their TRH test compared to group 2 (48.8% and 9.6%, p-value <0.001, **Table 1**). There was no age difference between the two groups (p-value 0.066, **Table 1**) and both groups consisted mainly of patients with non-hormonal secreting pituitary mass lesions (87.2% in group 1 and 58.8% in group 2, **Table 1**).

**Table 1:** Characteristics of the patients with thyroid replacement therapy started subsequently after a TRH test (group 1), and the patients with no thyroid replacement therapy started subsequently after a TRH test (group 2).

	Group 1	Group 2	P-values
Subjects, n	39	320	
Sex, m/f	27/12 (69.2%/30.8%)	145/175 (45.3%/54.7%)	0.005
Mean age ± SD	56.2 ± 17.9	50.6 ± 17.6	0.066
Diagnosis			
Pituitary mass lesion	34 (87.2%)	188 (58.8%)	<0.001
Hormonal producing adenoma*	4 (10.3%)	92 (28.8%)	0.014
Other	1 (2.6%)	40 (12.5%)	0.065
Surgery before TRH test	22 (56.4%)	62 (19.4%)	<0.001
Replacement therapies of other pituitary hormones before TRH test			
0	20 (51.3%)	289 (90.3%)	<0.001
1	15 (38.5%)	28 (8.7%)	<0.001
2	4 (10.3%)	3 (0.9%)	<0.001

P-values of comparison between group 1 and group 2. *Hormone producing adenoma includes prolactinomas, acromegaly and Cushing’s disease.

Comparison of results between the patients with thyroid replacement therapy started subsequently after a TRH test (group 1) and the patients with no thyroid replacement therapy started subsequently after a TRH test (group 2). T4 and TBG corrected T4 (T4_corr_) (mean, SD) and TSH (median, IQR) measured at baseline. TSH (median, IQR) measured 30 minutes after injection of 200 µg TRH i.v. Absolute response defined as stimulated TSH minus baseline TSH (median, IQR), and fold response defined as stimulated TSH divided by baseline TSH (median, IQR). P-values of comparison between group 1 and 2.

A comparable TSH_0_ was observed in group 1 and group 2 (p-value 0.824, **Table 2**). A significantly lower TSH_30_, TSH_fold_ and TSH_absolute_ was observed in group 1 compared to group 2 (p-value <0.001, <0.001 and <0.001, respectively). In group 1, 36 patients (92.3%) had decreased T4_corr_ measurements, and three patients (7.7%) had a T4_corr_ in the low normal range: 70 nmol/L-79 nmol/L.

**Table 2:** Characteristics of the patients with thyroid replacement therapy started subsequently after a TRH test (group 1), and the patients with no thyroid replacement therapy started subsequently after a TRH test (group 2).

	Group 1	Group 2	P value
Baseline T4, nmol/l ± SD	54.72 ± 14.81	81.03 ± 17.16	<0.001
Baseline T4_corr_, nmol/l ± SD	50.72 ± 14.88	82.01 ± 17.71	<0.001
Baseline TSH mlU/l ± IQR	1.50 ± 1.25	1.40 ± 1.10	0.824
TSH 30 min mlU/l ± IQR	5.40 ± 4.80	8.40 ± 7.22	<0.001
TSH absolute increase mlU/l ± IQR	3.70 ± 3.90	6.90 ± 6.37	<0.001
TSH fold increase ± IQR	3.30 ± 2.80	6.10 ± 3.60	<0.001

P-values of comparison between group 1 and group 2. *Hormone producing adenoma includes prolactinomas, acromegaly and Cushing’s disease.

### Subgroups of cases with decreased T4_corr_

**Figure 3:**
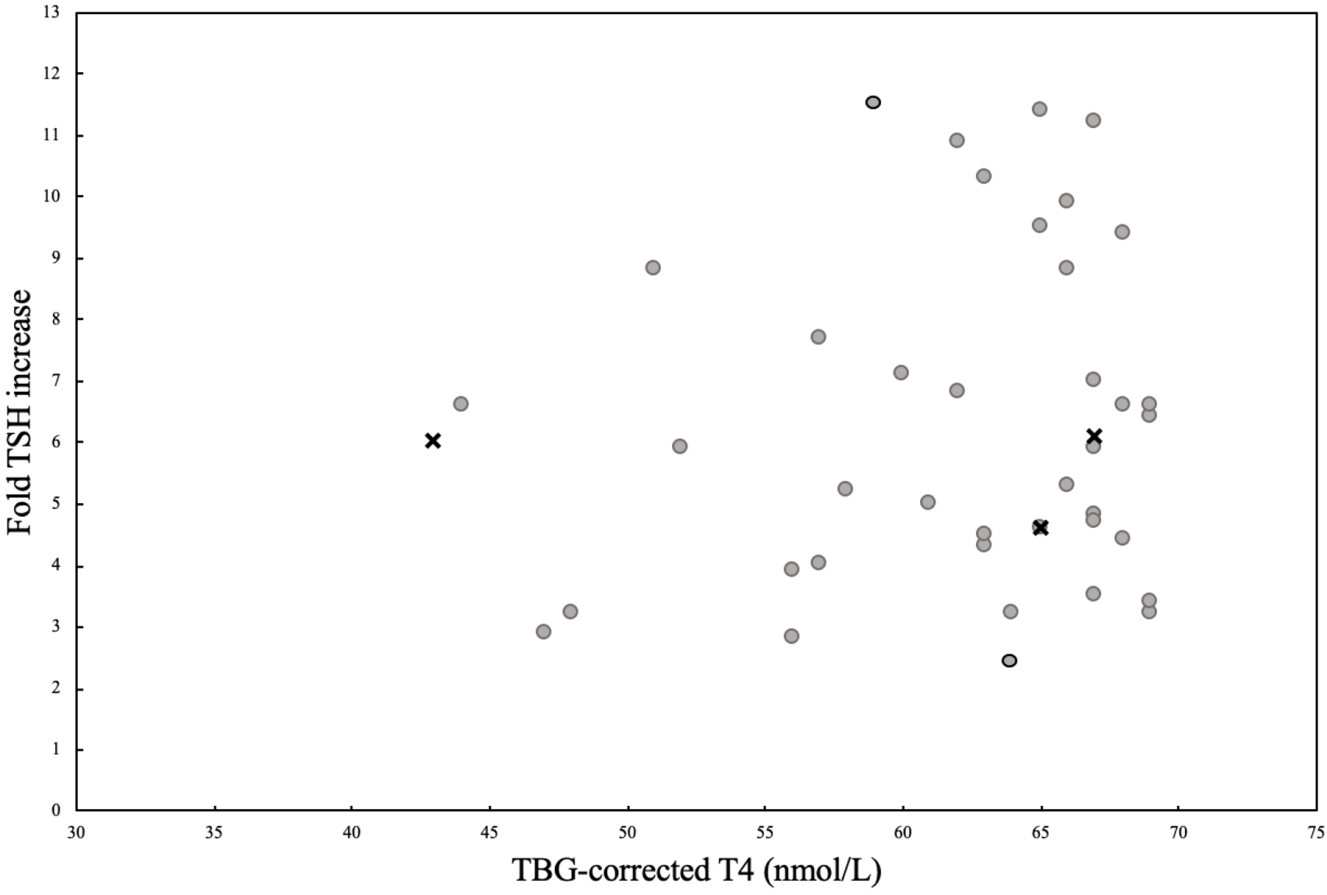
**(A)** A visualization of the patients from group 2A with TBG-corrected T4 values below the cut off level of 70 nmol/L, plotted according to their TBG-corrected T4 values and their fold TSH increase values. Group 2A (n=42) consists of the patients with the results of the TRH test interpreted as normal. Patients who never started thyroid replacement therapy are marked with grey dots (n=37). Patients who started thyroid replacement therapy later on, but had pituitary surgery between their TRH test and start of thyroid substitution therapy are marked with grey surrounded by black circle (n=2). Patients who started thyroid substitution therapy after spontaneously developing CH later on marked with crosses (n=3).

Patient characteristics for subgroup 1A, 2A and 2B including TRH test results are presented in **Tables 3**, **4** and **Figure 3**.

**Table 3:** Characteristics of the patients with a TBG corrected T4 < 70 nmol/L. Group 1A consists of the patients who received thyroid replacement therapy based on the results of their TRH test.

Patients with decreased TBG corrected T4 (<70 nmol/L)			
	**Group 1A**	**Group 2A**	**Group 2B**
Subjects, n	36	42	28
Sex, m/f	26/10 (72.3%/27.8%)	20/22 (47.6%/52.4%)	15/13 (53.6%/46.4%)
Mean age ± SD	57.2 ± 18.0	52.0 ± 16.6	53.2 ± 14.1
Diagnosis			
Pituitary mass lesion	31 (86.1%)	27 (64.3%)	21 (75.0%)
Hormonal producing adenoma*	4 (11.1%)	10 (23.8%)	7 (25.0%)
Other	1 (2.8%)	5 (11.9%)	0 (0.0%)
Surgery before TRH test	20 (55.6%)	9 (21.4%)	0 (0.0%)
Replacement therapies before TRH test			
0	18 (50.0%)	34 (81.0%)	28 (100.0%)
1	15 (41.7%)	7 (16.7%)	0 (0.0%)
2	3 (8.3%)	1 (2.4%)	0 (0.0%)
Symptoms			
No symptoms	7 (19.4%)	24 (57.1%)	21 (75.0%)
1 symptom	11 (30.6%)	12 (28.6%)	3 (10.7%)
2 or more symptoms	14 (38.9%)	6 (14.3%)	3 (10.7%)
No record	4 (10.1%)	0 (0.0%)	1 (3.6%)

Group 2A consists of the patients with TRH tests interpreted as normal and not started in thyroid replacement therapy after their TRH tests. Group 2B consists of the patients where other treatment was started with the intent of diminishing tumor size, e.g. surgery or dopamine agonist, and not started in thyroid replacement therapy subsequently after a TRH test. *Hormonal producing adenoma includes prolactinomas, acromegaly and Cushing’s disease.

**Table 4:** Comparison of results between the patients with a TBG corrected T4 < 70 nmol/L Group 1A consists of the patients who received thyroid replacement therapy based on the results of their TRH test.

Patients with decreased TBG corrected T4 (<70 nmol/L)					
	**Group 1A**	**Group 2A**	**P value**	**Group 2B**	**P value**
Mean baseline T4_corr_ nmol/l ± SD	48.75 ± 13.70	61.83 ± 7.07	<0.001	56.54 ± 8.04	0.010
Mean baseline T4 nmol/l ± SD	53.50 ± 14.68	73.24 ± 12.22	<0.001	62.00 ± 14.10	0.022
Median baseline TSH mlU/l ± IQR	1.45 ± 1.20	1.50 ± 1.66	0.414	1.10 ± 1.41	0.364
Median fold TSH increase ± IQR	3.50 ± 2.97	5.90 ± 3.75	<0.001	4.95 ± 1.88	<0.001

Group 2A consists of the patients with TRH tests interpreted as normal and not started in thyroid replacement therapy after their TRH tests. Group 2B consists of the patients where other treatment was started with the intent of diminishing tumor size, e.g. surgery or dopamine agonist, and not started in thyroid replacement therapy subsequently after a TRH test. TBG corrected T4 (T4_corr_) (mean, SD) and TSH (median, IQR) measured at baseline. Fold response defined as stimulated TSH divided by baseline TSH (median, IQR). P-values of comparison of group 1A with 2A and 2B.

In group 1A, a higher percentage of patients were treated with replacement therapies of other pituitary hormones than the thyroid axis at the time of the TRH test compared to group 2A (1A: 50.0% vs. 2A: 19.1%, p-value <0.001) and 2B (1A: 50.0% vs. 2B: 0.0%, p-value <0.001). The age at the time of examination and type of pituitary disease were comparable. A significantly lower level of T4_corr_ was observed in group 1A with CH compared to group 2A and 2B (p-value <0.001 and 0.010, respectively). TSH_0_ level was comparable between the three groups, but a significantly lower TSH_fold_ was observed in group 1A compared to group 2A or 2B (p-values <0.001 and <0.001, respectively). Significantly more patients reported >2 symptoms of CH in group 1A compared to group 2A and 2B (p-value 0.005 and 0.006, respectively), although 19.4% of patients in group 1A reported no symptoms suggestive of CH.

**Figure 4:**
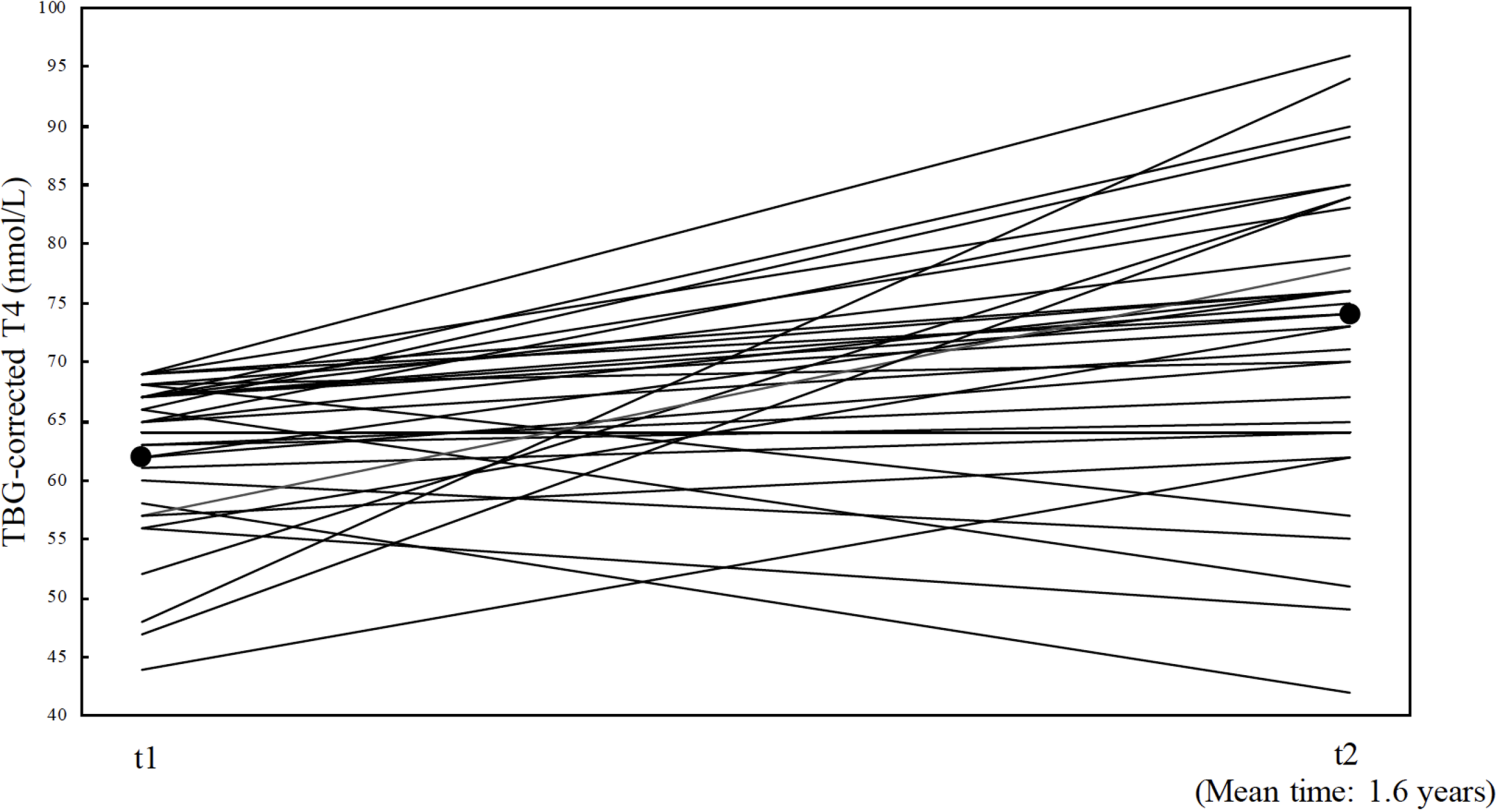
illustration of the 29 patients with follow up TBG corrected T4 from group T4 from group 2A (patients with TRH test interpreted as normal). Each patients is marked with a solid line, and represents their TBG-corrected T4 values at t1 (time of the TRH test) and TBG-corrected T4 values at t2 (their first follow up). Mean follow-up time are shown at t2 (1.6 years ±1.7). Mean TBG-corrected T4 values at t1(62 nmol/L ±7.4) and t2 (74 nmol/L ± 12.1) are marked with black dots.

In group 2A (n= 42), a total of 5 patients (11.9%) subsequently started thyroid replacement after a mean period of 2.9 years (0.7-6.7 years) from the TRH test. Of these 5 cases, 2 received pituitary surgery after the initial TRH test, but before the start of thyroid replacement therapy, whereas 3 cases (7.1%) spontaneously developed CH after a mean of 2.8 years ±1.4 years and 8 patients (19.0%) were lost in follow-up. At the time of the initial TRH test (t1), the remaining 29 patients showed a mean T4_corr_ of 62 nmol/L (SD ± 7.4). The mean time for the first follow-up (t2) was 1.6 ± 1.7 years where the mean T4_corr_ increased to 74 nmol/L ± 12.1 (p-value <0.001). At the last follow-up (t3) after 7.6 ± 5.0 years, T4_corr_ was significantly higher than t1 (p-value <0.001) with a mean T4_corr_ within the normal range of 73 nmol/L ±15.2 (**Figure 4**).

Group 2B consists of 23 patients (82.1%) awaiting pituitary surgery and 5 patients (17.9%) who started treatment with a dopamine agonist. A total of 10 patients (35.7%) who were all treated with pituitary surgery, from group 2B, were started on thyroid replacement therapy later, and 1 patient (3.6%) was lost in follow-up. The 17 patients (60.7%) in group 2B who never received thyroid replacement therapy showed a significant increase in mean T4_corr_ from 58 (SD ± 7.9) at t1 to 78 ± 19.8 nmol/L at t2 (mean time 1.5 ± 1.6 years, p-value <0.001). A further and significant increase was also observed at the last follow-up (after 8.7 ± 6.6 years) with a mean T4_corr_ of 75 ± 24.6 nmol/L (p-value 0.007). There was no correlation between the risk of developing post-surgical CH and the type of surgical procedures used or the need of post-surgical treatment including desmopressin or hydrocortisone replacement therapy (data not shown).

### Adverse effects

71.6% of the patients had registered mild side effects to the TRH test that disappeared spontaneously within minutes. These consisted of sweating (41.0%), palpitations (24.5%), dizziness (22.5%) and tiredness (16.3%), and were mainly observed at the TRH test accompanied by an ITT test. The side effects observed at the TRH test accompanied by a SAT were an urge to urinate (30.0%), nausea (16.6%) and a strange taste in the mouth (15.6%). One patient (0.3%) experienced a serious side effect consisting of a pituitary apoplexy in relation to the TRH test accompanied by a SAT. This patient was a 30-year-old male with a large prolactinoma with a size of 2.5x1.7x2.3 cm. He had a spontaneous remission without the need for surgery or hormonal replacement therapy.

## Discussion

TRH tests have routinely been part of pituitary stimulation tests performed for the diagnosis of pituitary insufficiency at the pituitary referral center at AalborgUH. A total of 359 individual TRH tests were performed, giving rise to a representative cohort of a wide range of pituitary adenoma cases. Each TRH test was interpreted in the respective clinical context, by one of three specialists in pituitary endocrinology who also oversaw the subsequent hormone replacement therapy. Based on this unique data, we observed that a large proportion of patients with a low T4_corr_ and normal fold TSH increase (TRH test) spontaneously normalized their T4_corr_ levels, whereas all patients with low T4_corr_ and low fold TSH increase were diagnosed with CH. We suggest that the TRH test could provide important information in selected cases presenting with low T4, normal TSH but without convincing signs of CH.

The diagnosis of CH is complicated due to varying bioactivity of circulating TSH and the rather unspecific symptoms of CH in patients with known severe diseases. Traditionally, the diagnosis of CH is based on a 1) low T4 level in conjunction with 2) a low, normal, or mildly elevated TSH 3) in the setting of pituitary disease. This usually confirms a CH diagnosis (5). However, in our cohort half of patients fitting this profile spontaneously normalized their T4_corr_ levels which were subsequently stable during long-term follow-up. Among the 106 patients (n=106) presenting with a decreased T4_corr_ measurement and TSH levels within normal range, only 36 cases (34%) were initially started on thyroid replacement therapy and a total of 41 cases (39%) were finally treated after long time follow-up where several cases had subsequently undergone pituitary surgery. The TRH test has been suggested to be a useful tool in the diagnosis of CH in cases with normal T4 (14–16). In our cohort only few patients with normal T4_corr_ were diagnosed with CH, however a pathological fold TSH increase was not observed in these cases. This may indicate that the TRH test should be restricted to patients with low T4, who do not present with convincing signs of CH.

The thyroid function spontaneously improved in most of the patients showing an initially low T4_corr_ and a normal TSH_fold_. After one year of follow-up, the mean T4_corr_ level had normalized, and this level persisted in the long-term follow-up 7.6 years with mean T4_corr_ values within the lower normal range. It is plausible that this small subgroup of patients accounting for only 12% of the total cohort (42 of 359) naturally would lie within the lower reference level of normal, and that even a small fluctuation in T4_corr_ could push this value below the lower reference values. All patients in this cohort were known to have a pituitary disorder, and it is well described that T3 and T4 decrease during acute or critical illness (17). In a group of critically ill patients, the T4 levels were reported to be reduced by 10-15% compared to healthy matched controls (17). Since we included only the first TRH test performed in each patient, we suspect that recent pituitary disease may be comparable to critical illness as regards the effect on pituitary thyroid function.

The baseline TSH measurement did not provide additional information in the diagnosis of CH, but in some patients an elevated TSH could confirm a diagnosis of primary hypothyroidism. The TSH levels were almost identical in all subgroups showing a low T4_corr_ and similar to persons with normal T4_corr_ level. In line with this, we did not observe a correlation between baseline TSH level and fold TSH increase (data not shown). Severe pituitary disease may serve as a marker of CH as significantly more patients with CH had known failure of other pituitary axes, i.e., multiple hormonal deficiencies. Symptoms of hypothyroidism also provide valuable information, although a large overlap in these and symptoms of chronic illness was observed (4, 7).

The strengths of this study lie within the high volume of TRH tests performed and the systematic use of tests in all patients at risk of developing hypopituitarism. Furthermore, all tests were evaluated by only few specialists in pituitary endocrinology, ensuring a high degree of uniformity in interpreting the test results. Finally, the follow-up period was rather long, showing stabilization and normalization of T4 levels in a large proportion of cases who initially presented with low T4 levels but normal TRH test results.

The retrospective nature of this study is its main limitation, due to the difficulty in assessing the decisive factor behind the diagnosis of individual cases. However, all clinical information is available including biochemical measurements, symptoms, the underlying diagnosis, and the applied treatment modality. Another limitation includes the inconsistency in follow-up and the lack of completeness of data. In particular, the data quality on the symptoms of CH is questionable since this is only sparsely recorded. Nevertheless, some inherent consistency should be expected since only a few highly specialized doctors were involved in the patient care. Since the TRH test was considered, an important factor in making the diagnosis of CH, the evaluation becomes somewhat self-fulfilling.

Our study suggests that the TRH test could provide important information mainly in patients with moderately decreased T4_corr_ (40-70 nmol/L). The TRH test could be restricted to a subgroup of patients accounting for less than 20% of our cohort. In patient with low T4 a TRH stimulated TSH increase less than 3-fold of the baseline TSH would suggest the diagnosis of CH whereas a more than 8-fold increase of TSH could rule out CH. However, in the remaining cases (TSH fold increase: 3-8), the diagnosis of CH could be supported by known hypopituitarism and symptoms of hypothyroidism. Importantly, a substantial proportion of patients with slightly decreased T4 seem to spontaneously regain normal pituitary-thyroid function.

## Data availability statement

The raw data supporting the conclusions of this article will be made available by the authors, without undue reservation.

## Ethics statement

Study was performed as a quality assurance study, and thus no ethical approval is needed, per local regulations. The studies were conducted in accordance with the local legislation and institutional requirements. The human samples used in this study were acquired from a by- product of routine care or industry. Written informed consent to participate in this study was not required from the participants or the participants’ legal guardians/next of kin in accordance with the national legislation and the institutional requirements.

## Author contributions

SC and LS: Joint first co-authorship. CR, HG, and KE: Assistance in data collection; revised the manuscript. PV: Critically revised the manuscript. EN: Provided TRH-tests; critically revised the manuscript. JK: Assistance with study design; critically revised the manuscript. JD: Study design, statistical assistance, critically revised the manuscript. All authors contributed to the article and approved the submitted version.
